# Health care professionals’ attitudes regarding palliative care for patients with chronic heart failure: an interview study

**DOI:** 10.1186/s12904-016-0149-9

**Published:** 2016-08-15

**Authors:** Jeanette Ziehm, Erik Farin, Katharina Seibel, Gerhild Becker, Stefan Köberich

**Affiliations:** 1 Section of Health Care Research and Rehabilitation Research, Medical Center – University of Freiburg, Faculty of Medicine, University of Freiburg, Freiburg, Germany; 2 Department of Palliative Care, Medical Center – University of Freiburg, Faculty of Medicine, University of Freiburg, Freiburg, Germany; 3 Pflegedirektion, Heart Center University of Freiburg, Freiburg, Germany

**Keywords:** Palliative care, Chronic heart failure, Interview study, Qualitative content analysis, Health care professionals’ attitudes

## Abstract

**Background:**

Even though struggling with similar symptom burden, patients with chronic heart failure (CHF) receive less palliative care than patients suffering from malignant diseases. Researchers have found that this might be related to lack of knowledge about palliative care, insufficient interprofessional communication as well as the cyclic course of disease which makes accurate prognosis difficult. However, research findings have shown that patients with CHF benefit from palliative care. As there are no studies for the German health care system this study aimed to assess health care professionals’ attitudes regarding palliative care of CHF patients in order to identify barriers and facilitators for this patient group and hence to develop recommendations for improvement of CHF patients’ access to palliative care in Germany.

**Method:**

Problem-centered interviews with 23 health care professionals involved in care of CHF patients (nurses: hospital, outpatient, heart failure, PC; physicians: hospital and resident cardiologists, general practitioners) were conducted and analysed according to Mayring’s qualitative content analysis.

**Results:**

Most interviewees perceived a need for palliative care for CHF patients. Regarding barriers patients’, public’s, and professionals’ lack of knowledge of palliative care and CHF; shortcomings in communication and cooperation of different professional groups; inability of cardiology to accept medical limits; difficult prognosis of course of disease; and patients’ concerns regarding palliative care were described. Different attitudes regarding appropriate time of initiation of palliative care for CHF patients (late vs. early) were found. Furthermore, better communication and closer cooperation between different professional groups and medical disciplines as well as better education about palliative care and CHF for professionals, patients, and public were cited.

**Conclusions:**

Palliative care for CHF patients is a neglected topic in both practice and research and should receive more attention. Barriers to palliative care for CHF patients might be overcome by: better education for the public, patients, and professionals, closer cooperation between the different professional groups involved as well as development of a joint agreement regarding the appropriate time to administer palliative care to CHF patients.

**Trial registration:**

DRKS00007119.

**Electronic supplementary material:**

The online version of this article (doi:10.1186/s12904-016-0149-9) contains supplementary material, which is available to authorized users.

## Background

Chronic heart failure (CHF) is one of the most common non-communicable diseases worldwide with an estimated prevalence rate of 2 % among the adult population [1]. Further, it is associated with high mortality rates [2]. Patients affected by CHF are suffering from multiple symptoms like dyspnoea [3], pain [4], and fatigue [5]. These symptoms lead to a reduction in physical capacity [5] and an impaired ability to perform activities of daily living [6, 7]. In consequence, patients with CHF, especially in an advanced stage of the disease, are dependent on the help of others [8].

According to the World Health Organisation palliative care refers to the improvement of quality of life of patients with life-threatening diseases by reduction and prevention of physical and psychological suffering through early identification and treatment [9]. Palliative care is not restricted to specific diagnoses but is mostly provided for oncological patients [10]. Even though symptom burden of CHF patients is as high as in patients with malignant diseases, data suggest that palliative care for CHF patients and patients with malignant diseases differs qualitatively as well as quantitatively [11, 12]. Reasons for the differences in the provision of palliative care for patients with malignant and non-malignant diseases are manifold, but could be mainly attributed to health care providers’ attitudes towards and their experience with palliative care [12–17]. Physicians’ and nurses’ perceived barriers to refer CHF patients to palliative care are based on the experience of poor interprofessional communication and collaboration [13, 15, 18] and divergent philosophies of different health care providers (cardiologist vs. general practitioner, nurses vs. physicians) [15, 17, 18], poor knowledge [13] about and insufficient skills to perform palliative care by general practitioners and cardiologists [13–15, 17], and difficulties identifying the appropriate time to refer CHF patients to palliative care [15, 16]. Furthermore, some health care providers think that there are not enough CHF patients to refer to palliative care [19] and that CHF patients do not want to discuss end-of-life issues [14–17]. A study conducted in Belgium found that general practitioners who are supposed to perform and coordinate primary palliative care only assessed acute care needs. As patients did not articulate non-acute palliative care needs to their general practitioner early identification of those needs barely happened [13].

Due to the mostly irreversible nature of CHF and the high symptom burden for patients and caregivers, access to palliative care services is recommended for these patients [20]. Further, research findings suggest that CHF patients benefit from palliative care as reflected in less drug use and hospitalisation rates as well as higher quality of life [21, 22]. Moreover, studies with patients with CHF revealed that patients and their caregivers need early support in order to better handle illness-related shortcomings, symptoms, and further proceedings [23, 24].

As health care systems differ with respect to provider education, responsibilities of the different health care providers, and organisation of care, data about experiences with and perceived barriers and facilitators regarding palliative care for CHF patients should be collected and analysed specifically for different countries. In fact, there are also differences regarding palliative care for patients with CHF and malignant diseases in Germany. For example, in 2014 only 3.3 % of all patients receiving palliative care were diagnosed with a cardiovascular disease as primary diagnosis [25]. Therefore, to add evidence to this field and to evaluate the situation within the German health care setting we conducted this study seeking to evaluate health care providers´ (nurses and physicians) attitudes towards and experiences with palliative care for patients with CHF in order to identify barriers and facilitators and hence develop recommendations for improvement of those patients’ access to palliative care.

## Methods

### Procedure

Problem-centred interviews [26] were conducted in order to collect experiences with and attitudes towards palliative care for heart failure patients. The recruitment of participants took place from October 2014 to March 2015. The recruitment area was characterized by a very good infrastructure regarding palliative care yet showing very few rates of CHF patients receiving palliative care. The goal was to conduct at least three interviews within each profession of interest (resident cardiologists, hospital cardiologists, general practitioners, outpatient nurses, hospital nurses, heart failure nurses) in order to cover the range of health care professionals usually involved in taking care of CHF patients. Professionals were chosen via existing contacts of two authors of the study (GB, SK) or by their entries in the phone book or internet. Participants were informed about the study (by telephone or e-mail) and asked to participate as an interviewee. After the participants gave informed consent, interviews took place face-to-face (83 %) or by phone (17 %) mostly at interviewees’ workplace. The interviews were conducted by one author (JZ) from January 2015 to April 2015. Interviews were audiotaped and later transcribed verbatim. The interviews lasted about 43 min each (*M* = 42.41, *SD* = 13.57).

The interview guide consisted of questions regarding the course of disease, care of heart failure patients at the interviewee’s workplace, experience with palliative care for heart failure patients, and palliative care of those patients in general (see Additional file 1). The guide was developed by a clinical nurse specialist for heart failure (SK) and a research assistant of the department of palliative care (KS) based on literature regarding this topic [2–6] and expertise. The interview was piloted in order to ensure comprehension and feasibility. Each interviewee was asked the main questions on the topic guide, with follow up questions used if required for clarification. The interviewer was also allowed to ask questions not shown in the interview guide if those would be necessary to clear content. Further, socio-demographic variables were obtained. The study was approved to be ethically and legally correct by the Ethics-Committee of the Albert-Ludwigs University Freiburg (vote number: 473/14).

### Sample

Altogether 23 health care professionals were interviewed (professions and their frequencies are shown in Table 1).

**Table 1:** Professions of Interviewees

Profession	Number (%)	Male (%)
Nurse (hospital)	4 (14)	0 (0)
Nurse (outpatient care)	3 (13)	1 (33)
Nurse (heart failure)	4 (17)	0 (0)
Nurse (PC)	1 (4)	1 (100)
Cardiologist (resident)	3 (13)	2 (67)
Cardiologist (hospital)	4 (17)	3 (75)
General practitioner	3 (13)	2 (67)
Other (referred to as palliative nurse 2)	1 (4)	0 (0)

Interviewees were on average 49 years old (*M* = 49.02, *SD* = 8.79) and more than half of them were female (61 %, for distribution of gender within the professions see Table 1). About three-quarters of participants (74 %) worked full time. The interviewees had been working with heart failure patients for about 20 years (*M* = 20.43, *SD* = 9.73). One of the general practitioners was trained in palliative care.

### Data analysis

Data was analysed according to Mayring’s [27] Qualitative Content Analysis in order to structure the collected data into categories and subcategories. With this approach, data can be reduced and categorised and thus systematic text processing is enabled. For this study the summary technique was used. The first step was to paraphrase the text by translating content into more simple and consistent short forms and to delete repetitions and vacuous passages. Then the paraphrases were generalised into more abstract levels. In the final step the generalisations were grouped into categories and thus a categorical system of the text was developed [27]. All interviews were analysed according to this procedure by one author (JZ); another author (SK) additionally analysed two interviews in order to enhance reliability of coding. The analyses were compared and adjustments in the coding system were administered if necessary. Moreover, three authors of this article (SK, KS, JZ) comprised an analysis group in which three interviews were expansively discussed in order to ensure validity of the procedure. Analyses were conducted using the programme Atlas.ti [28]. Interview parts portrayed within this article were translated from German into English (and slightly adjusted in order to preserve meaning) by one author (JZ) of this study.

## Results

In the following, a selection of the data derived category system is presented. We chose categories and subcategories which directly refer to palliative care for heart failure patients as the research question addresses professionals’ attitudes regarding palliative care for this patient group in order to improve CHF patients’ situation. Therefore, we concentrated on reasons for but also against palliative care for CHF patients as well as barriers, appropriate timing, and possible future directions. The overall category system is available in the supplements (see Additional file 2). Subcategories are illustrated in italics.

### Potential usefulness of palliative care for patients with CHF

This category groups reasons why palliative care for CHF patients would be useful. Interviewees from all integrated professions stated that a *need for palliative care for CHF patients exists* and that this *need increases* due to the growing prevalence of the disease. This goes in line with health care providers’ perception that *palliative care for this patient group makes sense*. In this respect it was mentioned that patients want palliative care because they want to avoid life-prolonging treatment which might reduce quality of life. Especially for older patients for whom specific therapies are not applicable anymore, palliative care was seen as proper treatment. Further, medical limits (CHF as incurable disease) as well as risks and side effects of invasive heart failure treatments were used to underline the perception that for some patients a *de*-*escalation of therapy would be more appropriate*.

‘It is a very important field which needs to be expanded. […] so that a CHF patient in a very bad condition, no matter what ending is going to come, has the possibility to get care in this field.’ (heart failure nurse 2)

‘I have the feeling that lots of patients want it [PC], they do not want their bodies to be connected to machines and devices until the end.’ (resident cardiologist 1)

‘Sometimes I wish that less therapy would be more therapy […] and that we can have more decisions in which we pursue a de-escalation of therapy instead of an escalation because life is terminal. We are all going to die someday.’ (resident cardiologist 1)

#### Potential uselessness of palliative care for patients with CHF

Especially in the group of cardiologists, palliative care for CHF patients was partly seen as *unnecessary because of the medical progress in the improvement and development of new therapies*. Accordingly, due to the progress of the last 20 years, applicable and functional therapies are available - also for older patients. Thus, therapy enables patients to handle their daily lives appropriately which in turn makes palliative care unnecessary. Further, some physicians took the view that the *need of palliative care for CHF patients is too small or non*-*existent* and therefore *only useful in certain cases*. Those interviewees stated that in their daily practice they experienced no or just a few patients suffering from CHF who would benefit from palliative care.

‘There was a dramatic change in the last 20 years. Now we administer extensive therapies in older groups which wasn’t done when I started in medicine.’ (hospital cardiologist 2)

#### Potential barriers to palliative care for patients with CHF

This category comprises barriers which complicate or hinder palliative care for CHF patients as perceived by the interviewees. All investigated professions perceived a *deficit in knowledge regarding content and structure of palliative care* for both professionals and patients. Further, it was believed that *patients might identify palliative care as euthanasia or assisted dying*. The impression that *palliative care only addresses cancer patients* often occurred.

‘I have the impression that palliative care in Germany is strongly tumour related thus far.’ (hospital cardiologist 1)

Another barrier was seen in interdisciplinary shortcomings such as *poor cooperation between professions and medical disciplines* or *competition for patients* as some physicians might fear the loss of patients to the palliative care unit. Generally, physicians of all subgroups (cardiologists and general practitioners) described *cardiology as a discipline which is not able to accept medical limits*. This means that cardiology is perceived as prolonging non-palliative treatment because palliative care is seen as defeat.

‘Often I don’t witness that stage, because the general practitioner takes care […] and often makes the decisions […] and often just continues his/her treatment.’ (resident cardiologist 3)

‘As a cardiologist you are taught very early that there is always a way and that everything can be done.’ (resident cardiologist 3)

The *unpredictable course of the disease* was judged as a further barrier because the appropriate time for the inclusion of palliative care is hard to determine because of the possibility of recurring phases of stability even after severe decompensations. In this regard it was also claimed that CHF patients *do not match required hospice admission criteria* as time of death is not foreseeable and thus the probability for CHF patients to receive hospice care is very low.

‘It is claimed that people in the last weeks or months of life can be accepted [for hospice stay]. […] Further, specific diagnoses are mentioned: terminal organ failure. […] it is hard to say when this stage is reached for CHF […] as our hospice is focusing on the actual end of life.’ (palliative nurse 1)

Doubts which (might) occur from patients’ perspectives were also perceived as barriers to palliative care. A lot of patients are said to be *not accepting of their mortality* and thus do not want that their active therapy to end and hence insist on *administering everything possible*. Further, it was stated that *patients are not aware of the severity of their disease*. Therefore, patients do not know that they suffered from a lethal disease because *nurses and physicians do not educate them completely* about the course of CHF. Another reason might be that the course of the disease with recurring phases of stability prevents patients from realising the severity of their illness.

‘It is not a huge topic for patients. They do not demand palliative care because when a patient gets that diagnosis he or she is not told: “you might have 4 years left” […] the doctors don’t make that clear.’ (hospital nurse 1)

#### Appropriate time for the initiation of palliative care for heart failure patients

In this category opinions regarding the right time for palliative care in the course of CHF are summarised. There was no clear consensus whether palliative care should be initiated early or late in the course of the disease between or within the interviewed professions. Arguments for *early palliative care* included better awareness and emphasis on quality of life as soon as possible. On the other hand, patients’ effectiveness in handling their daily lives as well as patients’ missing perception of a palliative situation were provided as reasons for a *late beginning* of palliative care.

‘A wish is to get the foot in the door earlier to raise awareness: “Ok, the basic situation cannot be changed but one can do a lot regarding quality of life or life expectancy” – without maintaining any empty promises.’ (general practitioner 1)

‘As long as patients can walk around, can keep their doctor’s appointments, I don’t think that they consider themselves to be in a palliative situation.’ (general practitioner 2)

Further, it emerged that the *nursing staff would initiate palliative care earlier than the physicians* do. In general, most of the interviewees were in favour of beginning palliative care in an advanced stage of the disease. This is *when all therapeutic opportunities are exhausted* or *when NYHA classification III or IV is reached*.

‘Often we see earlier that the patient is getting worse and until doctors and care unit have the same goal, it takes a long time sometimes. I think this is burdensome for me.’ (hospital nurse 1)

‘I would say it depends on the patient […]. For everyone at NYHA IV it is just definitely clear for me. Sometimes maybe already at the end of NYHA III.’ (heart failure nurse 2)

#### Expectations regarding future configuration of palliative care for CHF patients

In this category expectations or wishes for how future palliative care for CHF patients should take place or how medical disciplines and professions could cooperate in order to enable or improve palliative care for CHF patients are presented. For the latter, there was a strong wish for *closer cooperation between the medical disciplines* (cardiology, general medicine, palliative care, nursing staff). In this respect *intense communication and patient*-*centred meetings* between and within the professional groups were encouraged. Interdisciplinary case reviews or round tables as done in the treatment of cancer patients were seen as role models and thus could be transferred to the treatment of heart failure.

‘I think it would be very important to have good communication between care staff and physicians about a patient in special cases, maybe like a case meeting for each patient so the best possible conditions from therapy and palliative care can be obtained. So, there have to be a lot of conversations.’ (hospital nurse 2)

Another possible way to improve the situation for CHF patients was seen in a *palliative care unit functioning as consultant* for other professional groups. The palliative care unit should be available in need and should support the care staff in making decisions. Furthermore, some interviewees preferred the *integration of a palliative care unit into the hospital or the care units* so patients can stay in the same place while being treated palliatively, which might be better for the patients who are used to their treatment surroundings.

‘I wish it will develop in a cooperative manner. […] but me personally I would wish that [PC] would also be possible within a cardiologic clinic – I think it would be a pitty if we would hand it [PC] over completely.’ (hospital cardiologist 1)

Further, a huge *need for further professional education in palliative care* was expressed by all interviewed professional groups. Different forms of education were suggested, for example a palliative care unit introducing their opportunities to the care staff or multidisciplinary educational programmes in which every discipline/profession profits from each other. Another important topic in this regard was *public relations activity* in order to provide awareness for CHF as well as for palliative care for patients, care staff, and general public.

‘It is important I guess to promote it in the teams so they bring the people [PC staff] in, because they have more knowledge. So not everyone still bakes his/her own bread. […] it needs advertisement.’ (heart failure nurse 3)

## Discussion

This is the first study which has investigated health care professionals’ attitudes regarding palliative care for heart failure patients in Germany. The provision of palliative care for CHF patients was largly appreciated by the interviewees as a need for palliative care in this patient group was cited. Although the WHO recommends an early initiation, palliative care was mainly understood as end of life care by interviewees. According to Radbruch and Payne [10] end of life care can be regarded synonymous with palliative care but also more specifically as care in the last days or hours of life. In this study health care professionals tended to focus on the latter when referring to palliative care.

Regarding perceived barriers to palliative care for CHF patients, the results of international studies were replicated. Some physicians thought that there was no need for palliative care for CHF patients [19] as too few patients would benefit from it. Further, it was stated that patients do not accept their mortality [14–17] and hence do not want to be treated palliatively. Poor knowledge [13] about palliative care as applicable for patients with CHF was also described as a barrier as well as lack of cooperation and different point of views between the different professional groups involved in care of CHF patients [11, 13]. Delphi methods conducted within the German health care system regarding improvement of palliative care in general also found that closer cooperation between the professionals involved in care is necessary [29, 30]. Bekelman and colleagues [31] tested a collaborative care model for patients with CHF including palliative care gaining promising results.

Despite the shortcomings of professional cooperation, cardiology especially was described as a discipline which prolongs curative treatment as medical limits cannot be accepted. Previous research has also identified cardiologists’ procedures as barriers to palliative care as patients’ palliative care needs are not recognised, no holistic approach is practiced [15], and few cardiologists are discussing palliative care issues with patients even though being recommended by guidelines [32]. However, non-acceptance of medical limits was not identified as a barrier. Cardiologists’ training programmes should try to convey a more realistic picture about medical limits especially when it comes to non-communicable diseases, and palliative care should be covered in more depth.

There was no consensus regarding the appropriate time of initiation of palliative care within the course of disease but most interviewees preferred involvement of palliative care in an advanced stage. Further, interviewees stated the cyclic course of disease as a problem when deciding about involvement of palliative care. Some studies have described the difficulty of identifying the right time for commencement of palliative care for CHF patients due to the mostly unpredictable course of disease (e.g., [16]). Practitioners are afraid of lacking skills for discussing end-of-life or of discussing this topic too soon [15] or openly [24]. Here, tools which can help to identify CHF patients with palliative care needs might be useful. For example, use of algorithms which scan electronic patient records and forward matching patients to palliative care were found to triple consultation rates [33]. Further, development of criteria which facilitate identification of patients who could benefit from proactive palliative care seem fruitful [34]. Furthermore, existing methods like the Gold Standard Framework might be useful in the prediction of CHF patients’ course of disease [35]. Information about content and structure of palliative care could be given to patients at an early stage as this might facilitate care at a time when patients might benefit from it [36].

As a lot of barriers focused on lack of knowledge, it seems that there is a huge need for education about CHF and palliative care for professionals, patients, as well as the public. Education regarding palliative care seems to be crucial in order to overcome barriers to palliative care not only for CHF patients [29, 30]. In particular education of professionals regarding possibilities for palliative care of CHF patients should be intensified as patients could benefit from (early) admission to palliative care [21, 22]. For example, Kirolos and colleagues showed that referral rates to palliative care or hospice service increased through structured training of health care professionals regarding end-of-life care [37]. Joint case meetings for the different medical disciplines and professional groups might also be educational as exchange of knowledge can take place in an applied manner. Our study revealed that health care professionals perceived the public as not aware of the severity of the disease. They further perceived that the public strongly associated palliative care with immediate death or assisted dying. This was also found in previous studies, e.g., Japan [38] and Ireland [39]. Hence, educational programmes which create more knowledge and awareness about CHF and palliative care for the broader public (e.g., open-door days in clinics) are indicated.

Besides closer cooperation and more education, integration of palliative care into treatment for CHF was cited regarding future configuration, e.g., palliative care as part of hospitals or care units. Even though early involvement of palliative care into the treatment of CHF is recommended in most relevant guidelines (e.g., [1, 32]) CHF patients receive little palliative care treatment [9, 10]. Research suggests nursing staff could take over a pivotal role in coordinating palliative care between professionals as well as providing all relevant information to patients and relatives [40]. In this respect a contact person trained in palliative care might be available in each institution or care unit in order to implement palliative care in non-palliative care environments.

Some limitations need to be addressed. The study consisted of a local sample owing to practicability. Most interviewed health care professionals were recruited in areas which are characterised by an elaborate palliative care infrastructure, yet barriers to palliative care for CHF patients could be identified. As the interview study served the purpose of collecting information about health care professionals’ attitudes and experiences in order to develop a questionnaire for nationwide assessment, the sample size could be considered sufficient. By using a qualitative approach this study contributes to a broader understanding of health care professionals’ attitudes regarding palliative care for CHF patients. Using an open answer format enabled exploration of important topics which might have not have been covered with fixed answers in a questionnaire. Further, the broad spectrum of professional groups involved in the treatment of care for CHF patients (nurses in different areas, physicians of different disciplines) recruited in this study is a strength.

The results of this study were used to develop a questionnaire which was administered nationwide in order to assess health care professionals’ attitudes in a broader manner. The results of the questionnaire survey [41] as well as the interviews served as a foundation for a three-round Delphi method with experts and practitioners in the field of cardiology, palliative care, nursing, and health care administration (paper in preparation). Within this Delphi method, actual recommendations for practical implementation should be developed in order to overcome barriers for palliative care of CHF patients and to improve CHF patients’ access to palliative care [42].

## Conclusion

Palliative care for CHF patients is an important topic which should receive more attention in research and in practice. Findings that CHF patients benefit from palliative care should be taken seriously and thus should lead to increased accessibility of palliative care for this patient group. Educational programmes for public, professionals, and patients should be developed in a multiprofessional manner as appreciation of palliative care for CHF patients seems high while knowledge about it does not. This study contributes to better care for CHF patients as health care professionals’ attitudes regarding palliative care for CHF patients have been identified and actual recommendations for implementation in practical settings will be developed.

## Additional files

Additional file 1: Title of data: Interview Guide PaCa-HF Study. Description of data: Interview guide developed for the study and used as basis for each interview of the study. (DOCX 39 kb) Additional file 2: Title of data: Coding scheme. Description of data: Coding scheme derived from the interviews according to Mayrings’ Qualitative Data Analysis. (DOCX 43 kb)

## Abbreviations

CHF: Chronic Heart Failure
M: Mean
PC: Palliative Care
SD: Standard Deviation
