# Supplementary material for: Health care professionals’ attitudes regarding palliative care for patients with chronic heart failure: an interview study
Source: BMC Palliat Care. 2016 Aug 15;15:76. doi: 10.1186/s12904-016-0149-9 (PMC4986383; doi:10.1186/s12904-016-0149-9)
Supplement: Additional file 1: — Title of data: Interview Guide PaCa-HF Study. Description of data: Interview guide developed for the study and used as basis for each interview of the study. (DOCX 39 kb) [file 12904_2016_149_MOESM1_ESM.docx]

**Interview Guide PaCa-HF Study**

| **Part 1 – Opener: Introduction, experiences with heart failure patients, typical course of disease** | | |
| --- | --- | --- |
| **May you introduce yourself and your work shortly?**  **Today’s interview is about patients with chronic heart failure (CHF) and their care situation. If you think of one of your patients in particular, could you describe the typical course of disease from your point of view?** | | |
| **Content** | **Maintenance questions** | **Further questions** |
| - Course of disease - Own/professional view regarding disease and patients‘ needs | - What else comes to your mind? - Anything else? - What then? - And further? - What do you mean? |  |

| **Part 2: Taking care of CHF patients in the hospital or practice** | | |
| --- | --- | --- |
| **And now thinking about your workplace and the care of patients with CHF - could you please describe how patients suffering from CHF are taken care of at your workplace?** | | |
| **Content** | **Maintenance questions** | **Further questions** |
| - Personal goals of care | - What else comes to your mind? - Anything else? - What then? - And further? - What do you mean? | - On what focuses your care of patients with CHF? - What is important to you personally? |
| - Quality of life |  | - What role does quality of life play in your care? - Could you name an example? |
| - Needs - Agreement of therapy goals - Integration of relatives |  | - How do you assess patients‘ needs? - How do you agree therapy goals with the patients? - How do you integrate those goals into care? - How do you integrate relatives into care? Can you give an example? |
| - Dealing with exacerbation |  | - How do you deal with exacerbations in the course of disease? - In how far does patient’s age play a role in your decision? |
| - Cooperation with other professions |  | - With which professional groups of the health care system do you cooperate in order to take appropriate care of patients with CHF? - Who contributes which part of care? - How do you evaluate quality of cooperation? |
| - Quality of care |  | - How do you evaluate the current status of care of patients with CHF in general?   Optional:   - - What optimizations do you imagine?   - How can these optimizations be accomplished? |

| **Part 3: Interconnection of own work with palliative care (PC)** | | |
| --- | --- | --- |
| **Currently, approaches of joint care of CHF patients between cardiology and pc are developing worldwide. Can you please describe your experiences with specialized pc offers within the care of patients with CHF?**  **(In case specialized pc offers are not known, name them, e.g., specialized outpatient pc teams)** | | |
| **3a** | | |
| **Experienced with pc** | | |
| **Content** | **Maintenance questions** | **Further questions** |
| - Current cooperation/ personal part - Current structures of pc-treatment |  | - Can you describe the cooperation with xy more deeply? - Can you describe it with an example? - How did the cooperation start? - Do you know any further offers/structures of pc (which might be relevant for your work with patients suffering from CHF)? |
| - Experienced benefits and barriers | - What else comes to your mind? - Anything else? - What then? - And further? - What do you mean? | - What benefits did you gain from cooperating with xy? - Can you describe the benefits for your part of work/your work tasks in detail? - What influence did your work part had on the decision for pc? - From your point of view: What benefits did the patient gain from the cooperation with xy? - What kind of feedback did you get from patients/relatives regarding the additional pc at the beginning as well as at the end of treatment? - Was there something difficult or hindering for your work or for the patient? |
| - Attitude regarding future cooperation - Hopes and fears |  | - If there would be a closer cooperation with specialized pc in the future: What would define a good cooperation between cardiology and pc? - What would you hope for with regard on this kind of cooperation? - What kind of problems might occur? - Who should take over what tasks? - What kind of cardiological knowledge do employees of specialized pc services need in order to treat patients with CHF appropriately? |
| - Experiences and possible concerns of patients/relatives |  | - From the patient’s point of view: What kind of concerns might patients or relatives have regarding an additional pc? |

| **3b** | | |
| --- | --- | --- |
| **Not experienced with pc** | | |
| **Content** | **Maintenance questions** | **Further questions** |
|  | - What else comes to your mind? - Anything else? - What then? - And further? - What do you mean? | - Have you ever considered integrating an offer of specialized pc into your work? |
| - Content pc |  | - If **yes**   - What are the reasons for not having cooperated yet?   - Can you describe advantages of pc from your point of view?   - What do you think might be problematic or not good?   - What kind of further offers/structures of pc do you know which might be relevant for your work with patients suffering from CHF? - If there would be a closer cooperation in Germany between specialized pc and your workplace in the future: What would define a good cooperation between cardiology and pc?   - Who should take over what tasks?   - How could such a cooperation be initiated?   - At which point in the course of disease specialized pc should be involved?   - What would you hope for with regard on this kind of cooperation?   - What kind of problems might occur?   - What kind of cardiological knowledge do employees of specialized pc services need in order to treat patients with CHF appropriately? |
| - Actual barriers |  | - If **no**   - Why not? What did hinder you?   - Can you describe advantages of pc from your point of view?   - What do you think might be problematic or not good?   - What kind of further offers/structures of pc do you know which might be relevant for your work with patients suffering from CHF? - If there would be a closer cooperation in Germany between specialized pc and your workplace in the future: What would define a good cooperation between cardiology and pc?   - Who should take over what tasks?   - How could such a cooperation be initiated?   - At which point in the course of disease specialized pc should be involved?   - What would you hope for with regard on this kind of cooperation?   - What kind of problems might occur?   - What kind of cardiological knowledge do employees of specialized pc services need in order to treat patients with CHF appropriately? |

| **Part 4: Professional handling of mortality/limited life time due to disease** | | |
| --- | --- | --- |
| **When a patient with CHF is more and more exacerbating – how do you take care?** | | |
| **Content** | **Maintenance questions** | **Further questions** |
| - General pc by cardiological specialists/ resident cardiologists (definition, attitude, actual procedure) | - What else comes to your mind? - Anything else? - What then? - And further? - What do you mean? | - At which point in the course of disease of a patient with CHF do you change to pc? Can you give an example? - What does change through that switch in care? What kind of needs are focused then? Are there any important aspects in care? - Do you address the patient’s limited life time and favored care at the end of life to the patient/relatives? What facilitates or hinders that kind of conversation? - [Do you have inner barriers towards addressing end of life and issues?] - How prepared do you feel for conversations about end of life? - What are your experiences regarding patients’ reactions towards limited life time? - Are there any situations in which your care is limited? What kind of situations? How do you deal with them? |

| **Part 5: Closing – prognosis/solutions** | | |
| --- | --- | --- |
| **One last question: What is your prognosis: How will cooperation between cardiology and pc develop in the future?** | | |
| **Content** | **Maintenance questions** | **Further questions** |
|  | - What else comes to your mind? - Anything else? - What then? - And further? - What do you mean? | - What are you hoping for? - Can you imagine how a good cooperation could be achieved? |

| **Open question at the end** |
| --- |
| **Is there anything else you would like to add to this topic which was not part of the interview but might be important to you?**  **Thank you very much!** |
