# Supplementary material for: Health care professionals’ attitudes regarding palliative care for patients with chronic heart failure: an interview study
Source: BMC Palliat Care. 2016 Aug 15;15:76. doi: 10.1186/s12904-016-0149-9 (PMC4986383; doi:10.1186/s12904-016-0149-9)
Supplement: Additional file 2: — Title of data: Coding scheme. Description of data: Coding scheme derived from the interviews according to Mayrings’ Qualitative Data Analysis. (DOCX 43 kb) [file 12904_2016_149_MOESM2_ESM.docx]

| **Chronic Heart Failure** | **General Treatment CHF** | **Palliative Care CHF** | **End of Life** | **Future Directions** |
| --- | --- | --- | --- | --- |
| **Causes of Disease**   - Cardiomyopathy - Circulation related - Diverse - Flu - Some cause dramatic exacerbations - Valve diseases   **Comorbidities**   - Cholesterol - COPD - Depression - Diabetes - High risk of comorbidity - Multimorbidity - Patients’ Age   **Course of Disease**   - Creeping course - Creeping emergence - Cyclic - Depends on causes - Depends on (quality of) treatment - Diverse - Increase of hospitalisation - Prolonged - Prognosis - Severe - Stabilisation - Terminal diversity   **Diagnosis**   - Differential diagnostics - Follow-up monitoring - Valve disease easy to detect   **Quality of Life**   - Crucial - Differs individually - Examples - Importance for younger patients - Losses caused by therapy - Preconditions | **Boundaries**   - Cognitive impairments - Invasive therapy - Lack of medical supply - Lack of coping strategies - Lack of social support - Limited opportunities - Making decisions - Missing compliance - Missing relatives’ understanding - Patient attacks - Private issues - Psychological strains for staff - Psychological issues - Time factor - Too many patients - Unnecessary therapy escalation - Young patients   **Dealing with Exacerbation**   - Analysis of causes - Clarification of finances - Dialysis - Hospitalisation - ICU - Individual care - Inform doctor - Integration of PC - Living will - Offer remaining opportunities - Open approach - Patients’ requests - Psychological support - Recompensation - Symptoms of exacerbation - Transplantation, LVAD etc. - Validation of therapy   **Drug Treatment**   - Anticoagulation - Diuretics - Individual adjustment - Opiate for dyspnea - Rescue medicine - Wide range of medication   **General Treatment Practice**   - Better realisation of guidelines - CHF lobby too small - Cooperation with cardiologist and general practice not close enough - Differences between urban and rural regions - Hard to evaluate - Infrastructure needs to be improved - Insufficient number of resident - cardiologists - Insufficient number of care experts - Not good/ improvable - Little attention for patients - Positive - More financing - More PC - More psychological support - More staff - More telemedicine - More time - Need for individualised care - Optimisation possibilities - Optimisation possibilities – limits - Too much therapy - Under development/ improvement   **Interprofessional Collaboration**   - In-house very good - Quality improvable - With discharge management - With general practitioner - With hospital - Within the team - With medical assistants - With nursing home suboptimal - With nursing service - With other physicians, it is good - With other professions it differs - With other professions it is good - With others - With outpatient clinics - With physicians it is difficult - With physicians - With physiotherapists - With psychologists - With social workers - With spirituals   **Involvement of Relatives**   - Consideration of (socio)cultural differences - Dependent on relationship quality - Disease related - Education important - Less/none - Implications/problems for relatives - Important - Important in palliative situation - In case of patient request - Patients less open - Physicians do not have time - Support offers for relatives - Relatives differ regarding dedication - Requirements for relatives   **Needs Registration**   - Admission interview - Asking - Anamnesis - Care transition sheet - Documentation in care planning - Knowledge of patient - Needs - No standardised registration - Standardised - Via general practitioner - Via medical examination - Via medical tests - Via information - Via living conditions - Via medical round - Via relatives   **Outpatient Treatment**   - Aftercare via phone - CHF outpatient clinic - General practitioner - Outpatient area - Outpatient nursing service - Resident cardiologist   **Patients’ Age**   - Clarification if reanimation or IC is wanted when older - Death more acceptable when older - Dependent on communication - Plays no/minor role - Education is useful when younger elders take disease for granted - Exacerbation more acceptable when older - Impact differs individually - Impacts communication - Impacts medical aspects - More sensitive when younger - Mostly old patients - No impact on communication - Psychosocial aspects   **Requirements for Care Staff**   - Acceptance of mortality - Acceptance of patient’s decision - Forward knowledge to younger staff - Individual therapy - Personal resilience - Practicable therapy - Psychoeducation - Therapy suitable for everyday life   **Requirements for Patients**   - Acceptance of disease - Compliance - Keep in touch with general practitioner - None - Not to give up on self - Weight control daily   **Stationary Treatment**   - Cardiology station - Care regarding all options of modern medicine - Change-over for patients - Discharge management - Duration - Holistic care important - Hospice - Internal station - Procedure - Severe cases   **Therapy’s Main Focus**   - Adequate medical/nursing care - Companionship - Conversations - Diagnostics - Drug treatment - Education - Life style - Patients’ involvement - Patients’ needs - Patients’ relief - Regulation of hydration - Relationship of trust - Quality of life - Subjective parameters - Support organisation - Symptoms   **Treatment Aims**   - Agreement of medical consultation - Agreement with patients - Discharge management/ social workers are in charge - Documentation - Formulated at the end of stay - Formulated by patients - Formulated in treatment contract - Medical parameters - Not articulated - Optimisation of therapy - Resilience and well-being - Life support | **Advantages**   - Appreciation - Clarity regarding things that matter - Dignity - Escalation of therapy - Focus on prognosis - Focus on quality of life - Good that it is there - Holistic approach - Individual care - Intensive care - More time for patients - No negative aspects - Open approach regarding disease/death - Pain management/drugs - Preparation of critical situations - Relief for patients - Relief for physicians/nursing staff - Removal of fear of death - Spiritual care - Support of relatives - Tolerance   **Barriers**   - Appropriate time hard to determine due to course - Cardiologist/general practitioner conduct PC - Cardiology perceives PC as defeat - Care staff is not aware of prognosis - Course of disease seems unthreatening - Different professional approaches - Displacement of mortality - Easier to sustain therapy - Fear of relatives’ reactions - Hospice admission criteria not applicable for CHF - Intensive therapies applicable in older adults - Lack of PC beds/prolonged waiting time - Lack of communication - Lack of education by care staff - Lack of knowledge - LVAD/Transplant as life saver - Medical progress - No contact information - No financing - No/little need - No living will - Patients are overwhelmed - Patients’ age - Patients are not aware of prognosis - Patients do not demand PC - Patients do not get offers - Patients’ concerns - Patients demand therapy escalation - PC addresses cancer patients only - PC for CHF intangible - PC for CHF invisible - PC is identified as assisted dying - Relatives demand therapy escalation - Resistance of care staff - Therapy success enables daily living   **Definition of PC**   - Care of relatives as task - Care in an incurable condition - End-of-life care - Further tasks - Open communication/ relationship of trust - Protection from unnecessary therapies - Provision of care infrastructure - Provision of quality of life as task - Psychological care - Reduction of pain and suffering - Spiritual care   **Disadvantages**   - Bad pain management - Further disadvantages - Institutional contradictions in care - Stressful therapies until death   **Experience**   - Advanced training in PC - Already considered integration of PC - Already sent CHF patients to PC offers - Basic knowledge about PC - Benefit in general - Benefit for patients - Benefit for relatives - Benefit was small - Cooperation should be continued - Cooperation with PC was unproblematic - Feedback - Hospice group - Member in PC network - No knowledge of any other offers - Problems - Procedure cooperation PC - Procedure PC network - Realisation cooperation   **Facilitators**   - Actual need/growing need - Cardiology/general practice benefits from PC - CHF is growing problem - De-escalation of therapy more appropriate - Medical borders - Patients are grateful for help in palliative situations - PC for CHF patients useful - PC in individual cases useful - PC not hard to administer - Transplant/LVAD associated with risks/side effects   **Importance of Topic**   - Rewarding topic - Study raised awareness - Wonder why topic was neglected for so long   **Known Offers**   - Appropriate for CHF patients? - Bridge care for terminally ill cancer patients - Contacts available if needed - Department of PC - Hospice - Known outpatient offers - Little/no offers known - Nursing home - PC trained colleagues - PC trained general practitioners/PC network - PC work group - Specialised outpatient PC   **Necessary Cardiologic Skills**   - Acceptance of mortality - Assessment of prognostic exacerbation - Cardiologist should be involved - Cardiology training - Empathy - Experience with severe CHF - Experience with cardiologic patients - Handling of devices - Information about private problems/family from previous care - Medical background - No special training needed - Political ambition - Positive attitude - Symptomatic care competence - Trained in CHF (care experts/ intensive care) - Trained in PC   **No Experience**   - No consideration of PC integration for CHF patients yet - No outpatient offers known - No PC experience with CHF patients - No PC inpatient cooperation existent - No PC training - No transition for CHF patients to PC yet - Not involved in PC network - PC very limited availability   **Transition to PC (Desired Condition)**   - Always controversial in case patient does not wish for PC - Before terminal stage? YES - Before terminal stage? NO - Before terminal stage only in case of comorbidities - Early involvement of PC - Education about PC with diagnosis - Health problems while resting - Individual differences - Interdisciplinary problem - Interprofessional problem - Not before terminal stage - Nursing staff would involve PC earlier than physicians - Reasons for early involvement - Reasons for late involvement - Starting with NYHA III or IV - When patient’s wish changes - When possibilities of therapy are exhausted - When secondary diseases occur - When conventional therapy cannot provide quality of life   **Transition to PC (Status Quo)**   - As there is no curative approach for CHF all care is palliative - Depends on quality of life - In death phase - No mobility - Palliative patients should be respected like every other patient - When dialysis is needed - When patient refuses dialysis/treatment - When resources are exhausted | **Conversations about EoL**   - Addressing prognosis and limited life time directly - Collected experience while in training - Conditions - Conversation about EoL is part of treatment - Conversation about therapy vs. palliative - Conversations most of the time good - Coping through conversations with colleagues - Depends on diagnosis of CHF - Depends on relationship to patient - Employees are trained for conversations regarding EoL - Feeling badly prepared for conversations about EoL - Feeling prepared for conversations about EoL - Need for further education - No conversations regarding PC with CHF patients - No training for conversations - Patients’ age is relevant for conversation - Patients are grateful for openness - Patients’ social situation is relevant for conversation - Prepared through further education - Prepared through inner posture - Prepared through professional experience - Support of positive thinking/soothing of patients   **Facilitators Conversation about EoL**   - Advantageous when patients are true to themselves - A relationship of trust is advantageous - Clear familiar relationships - Consensus between every party involved - Illness insight - Involvement of general practitioner - Involvement of nursing staff and physicians is advantageous - Offer of help services - Openness/honesty - Presence of relatives - Proper cognitive ability of patient is advantageous - Sensitivity is essential   **Patients’ Reactions Regarding EoL**   - Depends on patients’ age - Different from cancer patients due to creeping course of disease - Displacement - Only few people accept mortality - Mostly positive - Patients are afraid of dying - Patients comply with guidelines - Patients do not want to take the situation anymore - Patients’ feelings when it comes to EoL - Patients know that they are terminally ill - Patients have lived their life - People recognise EoL earlier in outpatient settings - Patients who get things straightened out can die well - Patients who do not get things straightened cannot die well - Some people are blinded by belief in medicine or God - Some people cling to live - Very different   **Patient’s Request**   - Assessment of treatments which patients want to be done in terminal stages - Patients should decide who should continue treatment - Patients’ wish is more important than patients’ age - Talk about whether patients want to benefit from medical developments   **Place of Death**   - At home - Hospital - Nursing home   **Procedure**   - Address patients’ fears - Adaptation of medication - Cancel technical devices/life-prolonging treatment - EoL should be as comfortable as possible - In PC treatment focuses on symptoms - In PC whole team is involved - Involvement of ethics counselling - Involvement of pastoral care - Involvement of relatives - New goal setting in palliative situation - No break in the transition to PC - No treatment of fluid balance in EoL - Optimisation of possible heart surgery - Patient care is more intensive in palliative situation - PC works well - Palliative situation is not mentioned - Problems in actual procedure - Procedure, organisation, personal course are arranged in palliative situation - Protection of patients against anxiety/pain/dyspnoea at EoL - Psychosocial support - Relief for relatives - Resident cardiologist does not recognise treatment in terminal stage - Separate room - When conditions are appropriate patient stays at home in palliative situation   **Time of Conversation about EoL**   - At progressed stages - Early - Nursing staff informs physicians about conversation need for patients - When patient refers to the topic - When patients should start thinking about EoL | **Disadvantages/Problems of Cooperation between PC and Cardiology**   - Availability of PC - Both sides should not be over motivated - Bureaucracy - Data protection - Different attitudes of different professions - Mutual reservations - No time for further education - Nursing staff is more willing to cooperate than physicians - The more participants the more problems - Time of indication   **Ideal Cooperation between PC and Cardiology**   - Act in concert - Model oncological patients - At the beginning a lot can be done without specialised PC - Availability - Cardiology should not be supervised by PC - Close cooperation - Communication - Arrangements - Cooperation between inpatient and outpatient settings - Cooperation enables more professional care - Cooperatively - Coordinating unit - Counselling - Education in PC useful - Education regarding PC for CHF patients - Initiation of cooperation by PC - Involvement/education/ contact for patients - Main part should be carried out by nursing staff - More publicity of CHF and PC - Mutual observations/ introduction - No disadvantages in mind - Optimal care for patients - Organisational issues - Payment - PC as council - PC contact for nursing and medicine - PC might relieve general practitioner - PC network - PC should be implemented within the cardiology unit - PC should be implemented within the institution - PC should function as latent offer - Personal contacts - Prompt care - Regular (case) meetings - Supervision for staff - Transparency - Working groups   **Initiation of Cooperation between PC and Cardiology**   - Contract of cooperation - Further education - Generation of capacities/ financial funds - Initiation by PC - Involvement of organisations - Openness regarding initiation of cooperation - Pilot projects - Working group/project - Via PC unit within institution - Via regional networks   **Prognosis regarding Future Cooperation**   - Cooperation will grow - Need for improvement - No estimation/wish - Optimistic   **Role of Cardiology**   - Education of patients and relatives - Offer/forwarding of therapy in palliative situations - PC has to educate about care of CHF patients   **Role of PC**   - Cardiologic counsels - Cardiology as pretreating discipline - Cardiology should recognise   PC need and involve PC  evaluation of prognosis by cardiologist   - Cardiologist should take the lead in treatment - General practitioner should be take the lead in treatment - Patients should not be sent away to die - Resident cardiologist needs to be involved |
